# Altered Mesolimbic Dopamine System in THC Dependence

**DOI:** 10.2174/157015911795017083

**Published:** 2011-03

**Authors:** S Spiga, A Lintas, M Diana

**Affiliations:** 1 Dept. Animal Biol. and Ecology, Univ. of Cagliari, Italy; 2 “G.Minardi” Laboratory of Cognitive Neuroscience, Dept. of Drug Sciences, Univ. of Sassari, Sassari, Italy

**Keywords:** Cannabinoids, dopamine, nucleus accumbens, withdrawal.

## Abstract

To explore the functional consequences of cannabinoid withdrawal in the rat mesolimbic dopamine system, we investigated the anatomical morphology of the mesencephalic, presumed dopaminergic, neurons and their main post-synaptic target in the Nucleus Accumbens. We found that TH-positive neurons shrink and Golgi-stained medium spiny neurons loose dendritic spines in withdrawal rats after chronic cannabinoids administration. Similar results were observed after administration of the cannabinoid antagonist rimonabant to drug-naïve rats supporting a role for endocannabinoids in neurogenesis, axonal growth and synaptogenesis. This evidence supports the tenet that withdrawal from addictive compounds alters functioning of the mesolimbic system. The data add to a growing body of work which indicates a hypodopaminergic state as a distinctive feature of the “addicted brain”.

## INTRODUCTION

Clinical reports described that chronic consumers of even low daily doses of cannabis derivatives, experience upon cessation of drug administration, overt abstinence signs [1, 2]. These findings are paralleled by structural and functional neuroimaging studies of cannabis use [3]. Likewise, overt somatic signs of cannabinoid withdrawal can be elicited in experimental models of cannabinoid dependence by administering the competitive antagonist of cannabinoid CB_1 _receptors rimonabant (SR) [4-7], thereby offering the possibility to investigate neurobiological effects of cannabinoids in a condition that mimics human addictive behaviour [8, 9].

The morphological analysis of neurons [10, 11] has recently seen a widespread increase of the studies concerning consequences of long-term administration of drugs [12-15]. In fact, these measures are likely to reflect plasticity of active synapses and, therefore, synaptic remodelling as a consequence of experience and/or drug-exposure [12]. In this regard, addiction has been conceptualized as one example of experience-dependent plasticity whereby experience (i.e. long-term exposure to addictive drugs) may affect behavioural, cognitive and psychological functions in a long lasting way [12, 16].

Cannabinoid withdrawal produces a marked reduction of electrophysiological activity in NAcc-projecting dopamine (DA) containing neurons of the rat midbrain [4] and a reduction of DA outflow in the NAcc shell [17]. This functional evidence suggests that cannabinoids withdrawal may structurally alter cellular elements of the mesolimbic system, as it was recently shown for opiate dependence [14, 15, 18]. Accordingly, chronic Δ^9^ TetraHydroCannabinol (THC) treatment has been shown to increase the length of the dendrites as well as the number of dendritic branches in both the shell of the NAcc and the medial prefrontal cortex, though not in other brain areas [19]. However, since morphological evaluations were made long after drug discontinuation (30 days), it is impossible to ascertain the relationship between onset of withdrawal and structural changes.

Thus, in the present study we investigated the morphological alterations of Ventral Tegmental area (VTA) and Substantia Nigra pars compacta (SNc) DA neurons and their accumbal post-synaptic counterparts, the spiny neurons (MSN). TH-positive neurons and Golgi-Cox stained MSN were evaluated after chronic cannabinoid treatment and withdrawal in order to obtain further insights into the morphological features of the mesolimbic DA system and its involvement in cannabinoid dependence. In addition, the role of endogenous cannabinoids was investigated through administration of SR to drug-naïve rats.

## MATERIALS AND METHODS

Male Sprague-Dawley albino rats (n=48; Charles River, Como, Italy), weighing 200-225 g at the beginning of treatment, were used. Rats were kept on a 12-h light/12-h dark cycle with food and water available *ad libitum*. Experimental protocols were approved by the Ethical Committee of the University of Sassari and performed in strict accordance with the EC regulations for the use of experimental animals (CEE N°86/609), and recommended guidelines for the care and use of experimental animals approved by the Society for Neuroscience. Rats were administered twice daily (08:00 a.m. and 20:00 p.m.), for 6.5 days with either Δ^9^-THC (Sigma, Milano, Italy) or CP 55,940 (Sigma-Aldrich, Milan, Italy) emulsified in 1% Tween 80, then diluted in a saline solution and administered i.p. in a volume of 3 ml/Kg.

Animals were assigned to the following groups: Chronic saline (Sal) (n=6); Chronic THC (15 mg/kg) (THC-C); 24 hrs spontaneous withdrawal from chronic THC (THC-W); precipitated withdrawal from chronic THC (15 mg/kg/administration) (THC-SR) (n=6); SR (5 mg/kg) in Sal (SR) (n=6) (6). On the morning of day 7 rats received the first daily administration (vehicle, CP or THC) and 1 hour before sacrifice were tested for signs of spontaneous and SR precipitated-withdrawal. Animals were anaesthetized with urethane (1.3 g/kg i.p.) before transcardiac perfusion with 100 ml of ice-cold saline solution immediately followed by 400 ml of ice-cold 4% paraformaldehyde. Brains were divided into two parts at -2 mm from bregma approximately.

The posterior part of brains, VTA- and SNc-containing, was post-fixed for 24 hours in the 4% paraformaldehyde solution and cryoprocteted in 30% sucrose in phosphate-buffered saline (PBS). Coronal sections between -5.80 mm and -6.30 mm (25 µm thick) from bregma, according to Paxinos and Watson [20] were obtained with a cryostat (Micron Cryo-Star HM 560, Walldorf, Germany). Sections for TH-immunolabelling were washed for 3x5 min in PBS, immersed for 30 min in 10% normal goat serum (NGS) in 0.1 M PBS added with 0.5% Triton X-100 (PBS-TX) and incubated for 2 hours with a mouse monoclonal anti-TH antibody (1:500; Chemicon, Temecula, CA, USA) in PBS-TX. Sections were then washed (3x5min) in PBS-TX and incubated with a biotinylated anti-mouse IgG (1:300, Vector Laboratories, Burlingame, CA, USA) in PBS-TX and 1% NGS for 30 min, rinsed (3x5 min) in PBS-TX and incubated with avidin-TRITC (1:200, Sigma-Aldrich, Milano, Italy) in PBS-TX and 1% NGS overnight at 4°C. All Sections were then washed (3x20 min) in PBS-TX and coverslipped with Glycergel mounting solution (Dako, Milano, Italy).

After perfusion, the anterior part of the brains, NAcc-containing, were immediately rinsed (15 min x 3 times) in 0.1 M PBS and immersed in a Golgi-Cox solution. The solution was changed once after 2 days and the brains were then left in fresh Golgi–Cox solution for additional 14 days. After this period the brains were cryoprotected with a 30% sucrose solution for 2-3 days. 50 µm thick coronal slices, beginning at 1.70 mm and ending at 0.70 mm from bregma, according to Paxinos and Watson [20], were obtained with a cryostat. Slices were developed using the procedure described by Kolb and McClimans [21].

Leica 4D Confocal Laser Scanning Microscope (CLSM) with an argon-krypton laser were used to analyse TH-positive neurons and Golgi-Cox stained sections. Confocal images were generated using 40x oil (na=1.00-0.5) and 100x oil (na=1.3). Each frame was acquired eight times and then averaged to obtain noise-free images. Optical sections, usually at consecutive intervals of 0.5 µm in Z axis, were imaged through the depth of the labelled neurons and saved as image stacks as previously described [13, 14]. *Maximum intensity* algorithm was used for 3D recostructions of TH-immunolabeled cells, while *Extended focus* algorithm was used for 3D recostructions of Golgi-Cox stained neurons (Bitplane Imaris V.7).

Morphometric analyses were performed by two independent observers blind to pharmacological treatments. TH-immunolabelled somata (80/group) were collected from a square area (approx. 200 µm/side). When totally included in the sections, TH-positive neurons were three-dimensionally reconstructed and used for measurements and statistical analysis using Bioscan Optimas software (version 6.5.1). The cell bodies were manually marked following their profile, excluding all dendritc trunks to measure their area (µm^2^).

For each treatment dendritic 80/group segments (at least 20 µm long) of second order dendrites were collected for analysis, from 0.7 to 1.70 mm from bregma (58) and identified by confocal rendered cells. Spines’ density was calculated by tracing a 10-15 µm long “sp” line, along the dendritic trunk and counting the number of spines therein. The procedure was repeated along the entire dendritic length from the bifurcation from the first branch of primary dendrites to the next bifurcation.

Data was processed by a one-way ANOVA followed by post-hoc comparisons using the Bonferroni criterion.

## RESULTS

**Fig. (1).**
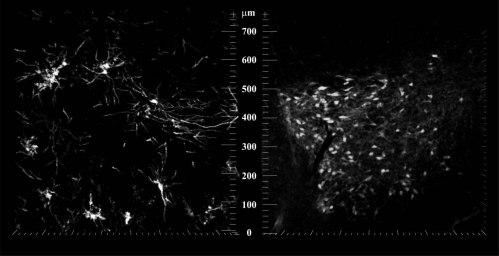
Confocal pictures of representative MSN Golgi stained in Nacc shell (left) and TH-positive neurons in the VTA in saline rats.

**Fig. (2).**
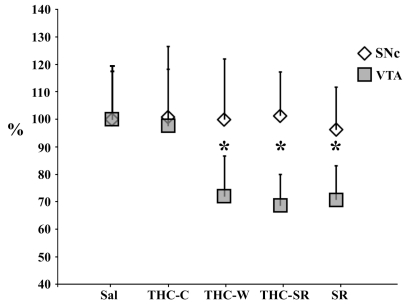
Percentage (respect of sal group) of mean ± SEM of VTA TH-positive neurons areas* indicates p<0.05 vs. sal.

Confocal datasets of TH-immunolabeled neurons located in the VTA (Fig. **1**) and in the dorsomedial portion of SNc [22] were morphometrically analysed in order to evaluate the effects of treatments. Analysis of variance showed anatomical differences for mean calculated area (F _639_=82.83; P<.0001), among experimental groups in the VTA. Post hoc analysis revealed that cell bodies in the VTA exhibited smaller somata under both THC withdrawals. In particular, a mean reduction was found for THC-W (t_158_= 10.9; P<0.0001) and THC-SR (t_158_= 7.22; P<0.0001), when compared with control. Surprisingly, we also found qualitatively similar changes in the SR group (t_158_= 12.1, P<0.0001) whereas no changes were observed in THC-C group (t_158_= 0.70, P= 0.48). No statistical differences were found for TH-positive neurons from SNc (F _639_=0.58; P=0.77), (Fig. **2**). 

**Fig. (3).**
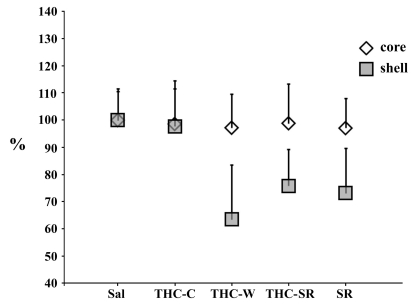
Percentage (respect of sal group) of mean ± SEM of dendritic spines densities (number of spines/10 µm of second order dendrites) of NAcc shell and core MSN. * indicates p<0.05 vs. CTRL (student t test post hoc analysis).

One way ANOVA revealed a significant effect in the shell (F _639_=107.2; P<.0001), but not in the core (F _639_=0.58; P=0.77), on spine density in the experimental groups (Fig. **3**).

Post hoc analysis, in the shell, showed a selective reduction in spine density for spontaneous THC-W (t_158_= 14.4 P<0.0001) and pharmacologically precipitated withdrawal (THC-SR t_158_= 12.6 P<0.0001) vs Sal. Interestingly, a reduction in spine density was also found in the SR group when compared to control (t_158_= 12.2; P<0.0001). Further, post hoc analysis failed to reveal any significant difference between spine density counts in the shell MSN for THC-C (t_158_= 1; P=0.32) groups vs saline treated rats.

## DISCUSSION

The present study shows that withdrawal from a regimen of chronic cannabinoid administration profoundly affects the morphological characteristics of TH-positive neurons of the rat VTA and dendritic spine density of MSN in the NAcc shell. In contrast, the area of SNc TH positive neurons, as well as core spine density, was unaffected.

In particular, spontaneous and SR precipitated withdrawal from chronic administration of Δ^9^ TetraHydroCannabinol, determined the most significant shrinkage of the soma of TH-positive neurons of the VTA. These changes were paralleled by a reduction of spine density in the secondary dendrites of accumbal MSN in the shell. In contrast, chronic administration of THC does not seem to affect the morphology of VTA neurons and spine density in the accumbal core MSN, thus pointing to a critical role of cannabinoids withdrawal in the shrinkage of mesencephalic neurons and spine loss in the NAcc shell. This further strengthens the view that withdrawal from chronic cannabinoid administration exerts powerful and long lasting [19] changes in key brain structures affected by addictive compounds.

The synaptic rearrangement described in the present study, is in line with, and significantly extends, previous findings [18] and could also be a key factor in the down regulation of CB1 receptors after THC-withdrawal [23], as well as the CB1-mediated inhibition of excitatory synaptic transmission at the excitatory synapses between the prefrontal cortex and the NAcc [24].

Interestingly, administration of the CB1 antagonist rimonabant in drug naive rats produced effects qualitatively similar to those observed in subjects treated with exogenous cannabinoids in the areas examined. This unexpected finding suggests that endogenous cannabinoids might be involved in the trophic control of key elements of the mesolimbic system such as VTA DA neurons and their physiological post-synaptic counterparts (i.e. MSNs). While further experiments are needed to corroborate this hypothesis, the present finding supports the idea of an endocannabinoid trophic and protective role [25, 26] at the level of the DA system [27]. Accordingly, endocannabinoids modulate synaptic plasticity in the VTA [28] and Substantia Nigra pars reticulata [30]. Alternatively, rimonabant might be acting as an inverse agonist [31, 32] and, therefore, further experiments will clarify this issue.

Irrespective of the mechanisms underlying our observations, the structural changes occurring at both pre- and post-synaptic levels are likely to have profound consequences on dopaminergic transmission in the shell of the NAcc. Indeed, the reduced dopamine firing [5] is accompanied by a “shrinkage” of the somatic region, thereby rendering the cell more excitable in line with the “size principle” [33]. At the post-synaptic side the reduced number of spines, besides the obvious loss of connections, would reduce total membrane surface [34, 35], thereby decreasing membrane resistance [36], eventually leading to altered excitability. This possibility would be in line with classical theoretical predictions, and subsequent confirmatory experimental tests [36], which ascribed to the spine an attenuating effect on synaptic potentials. Importantly, recent studies [37] employing a chronic regimen of THC very similar to the one employed here have reported that long-term exposure and subsequent withdrawal (recordings were performed 24 hours after last treatment) of THC, blocks synaptic plasticity in the NAcc and reduces the sensitivity of GABAergic and glutamatergic synapses.

Dendritic spines are particularly important in synaptic plasticity in reason of their rapid changes in volume and/or shape in response to stimuli. Is it true that spine number is very variable and they can spontaneously appear or disappear, but, what could then be the functional consequence of remodelling or losing about 30% of the dendritic spines? There are at least two mechanisms directly related to the reduction in the number of synapses, which can affect the overall firing rate of the neuron. The reduction of neuronal membrane associated with the loss of spine increases the input resistance of the neuron and, in principle, results in a more excitable neuron. On the other hand, the neuron may decrease its firing rate because the overall excitatory input is reduced.

Overall, the present data suggest that the altered architecture of the DA system projecting to the shell of the NAcc documented here would profoundly alter the synaptic equilibrium affecting various neurotransmitters involved in the neurobiological mechanisms of cannabis dependence [8, 16].

On the basis of the present and previous findings [14, 15, 18] we suggest that shrinkage of DA neurons and reduction of spine density on their post-synaptic elements (i.e. MSN) upon withdrawal from chronic cannabinoid administration might represent a morphological correlate of the functional deficits detected by electrophysiological [4] and neurochemical [17] means, which may ultimately contribute to negative motivational properties of withdrawal from addictive drugs [16, 37, 38].

In general, the present data lends further support to the notion that drug addiction can be seen as a chronic drug-induced, aberrant, form of neural plasticity [8, 12, 16, 38], whereby DA neurons originating in the VTA represent major cellular substrate involved at molecular, cellular [8, 39] and behavioral levels [38, 41-43] and is coherent with a recent hypothesis [16] underscoring a hypodopaminergic state as a distinctive feature of the “addicted brain”.
